# Identification of Intracellular and Plasma Membrane Calcium Channel Homologues in Pathogenic Parasites

**DOI:** 10.1371/journal.pone.0026218

**Published:** 2011-10-14

**Authors:** David L. Prole, Colin W. Taylor

**Affiliations:** Department of Pharmacology, University of Cambridge, Cambridge, United Kingdom; Univ. Georgia, United States of America

## Abstract

Ca^2+^ channels regulate many crucial processes within cells and their abnormal activity can be damaging to cell survival, suggesting that they might represent attractive therapeutic targets in pathogenic organisms. Parasitic diseases such as malaria, leishmaniasis, trypanosomiasis and schistosomiasis are responsible for millions of deaths each year worldwide. The genomes of many pathogenic parasites have recently been sequenced, opening the way for rational design of targeted therapies. We analyzed genomes of pathogenic protozoan parasites as well as the genome of *Schistosoma mansoni*, and show the existence within them of genes encoding homologues of mammalian intracellular Ca^2+^ release channels: inositol 1,4,5-trisphosphate receptors (IP_3_Rs), ryanodine receptors (RyRs), two-pore Ca^2+^ channels (TPCs) and intracellular transient receptor potential (Trp) channels. The genomes of *Trypanosoma*, *Leishmania* and *S. mansoni* parasites encode IP_3_R/RyR and Trp channel homologues, and that of *S. mansoni* additionally encodes a TPC homologue. In contrast, apicomplexan parasites lack genes encoding IP_3_R/RyR homologues and possess only genes encoding TPC and Trp channel homologues (*Toxoplasma gondii*) or Trp channel homologues alone. The genomes of parasites also encode homologues of mammalian Ca^2+^ *influx* channels, including voltage-gated Ca^2+^ channels and plasma membrane Trp channels. The genome of *S. mansoni* also encodes Orai Ca^2+^ channel and STIM Ca^2+^ sensor homologues, suggesting that store-operated Ca^2+^ entry may occur in this parasite. Many anti-parasitic agents alter parasite Ca^2+^ homeostasis and some are known modulators of mammalian Ca^2+^ channels, suggesting that parasite Ca^2+^ channel homologues might be the targets of some current anti-parasitic drugs. Differences between human and parasite Ca^2+^ channels suggest that pathogen-specific targeting of these channels may be an attractive therapeutic prospect.

## Introduction

Parasitic diseases collectively affect billions of people and cause millions of deaths worldwide each year (**Table 1**). Many parasitic diseases are caused by single-celled protozoa such as various species of the apicomplexan parasites *Plasmodium*, *Toxoplasma*, *Cryptosporidium* and *Babesia*, as well as several species of the kinetoplastid *Trypanosoma and Leishmania* parasites. Other parasitic protozoa that are major contributors to worldwide disease include *Trichomonas vaginalis*, *Entamoeba histolytica* and *Giardia intestinalis*. Another major cause of human disease and mortality, second only to malaria amongst parasitic diseases, is the *Schistosoma* family of parasitic blood flukes. Many of these parasites maintain stringent control over their intracellular Ca^2+^ concentration [1], [2] and have been shown to exhibit Ca^2+^ signals in response to physiological stimuli. These Ca^2+^ dynamics are critical for parasite function, egress, invasion, virulence and survival [2]–[4]. However, the molecular basis for these parasite Ca^2+^ responses is largely unknown.

**Table 1:** Pathogenic parasites with completed whole-genome sequences, their associated diseases and worldwide disease burden.

Parasite	Disease	Estimated worldwide cases of infection	Genome References
*Plasmodium falciparum Plasmodium knowlesi Plasmodium vivax*	malaria	3 billion [103], [126]	[127]–[130]
*Toxoplasma gondii*	toxoplasmosis	6–75% of population [131]	[132]
*Cryptosporidium hominis Cryptosporidium muris Cryptosporidium parvum*	cryptosporidiosis, diarrhoea	1–20% of population [131], [133]	[134]–[136]
*Babesia bovis*	babesiosis	humans: >500cattle: 400 million	[137]
*Leishmania major Leishmania infantum Leishmania braziliensis*	leishmaniasis	12 million [131]	[138]–[140]
*Trypanosoma brucei*	african trypanosomiasis (sleeping sickness)	500,000	[141]
*Trypanosoma cruzi*	Chagas' disease	10 million	[142]
*Entamoeba histolytica*	amoebiasis, dysentry	50 million [131]	[143]
*Giardia intestinalis*	giardiasis	280 million [144]	[145]–[148]
*Trichomonas vaginalis*	trichomoniasis	174 million [149]	[150]
*Schistosoma mansoni*	schistosomiasis (bilharzia)	200 million	[121]

World Health Organization (http://www.who.int/en) and Center for Disease Control (http://www.cdc.gov) figures, in addition to the references cited, were used as sources of worldwide epidemiology.

Ca^2+^ release via intracellular Ca^2+^ channels controls numerous cellular processes, ranging from receptor signalling to growth and apoptosis [5]. Four main families of these channels have been identified in mammals: inositol 1,4,5-trisphosphate receptors (IP_3_Rs), ryanodine receptors (RyRs), two-pore channels (TPCs), and some transient receptor potential (Trp) channels. Mammalian IP_3_Rs and RyRs are responsible for release of Ca^2+^ from endoplasmic reticulum (ER) in response to the intracellular messengers IP_3_ and Ca^2+^ (IP_3_Rs), or cyclic ADP ribose (cADPR) and Ca^2+^ (RyRs), produced via a wide variety of cellular signalling pathways [6], [7]. The recently described TPCs are nicotinic acid adenine dinucleotide phosphate (NAADP)-activated channels responsible for Ca^2+^ release from acidic organelles, including lysosomes [8], [9]. Mammalian intracellular Trp channels such as TrpM, TrpML and TrpP2 (polycystin-2) have also been shown to play a role in release of Ca^2+^ from intracellular stores [10], [11]. Many parasites possess intracellular Ca^2+^ stores within their ER, mitochondria, glycosomes and acidocalcisomes [2], [3], [12], [13], but whether channels homologous to mammalian intracellular Ca^2+^ channels are present in these parasite organelles and whether they mediate Ca^2+^ release are unknown.

Ca^2+^ entry in mammalian cells is mediated by plasma membrane Ca^2+^ channels such as voltage-gated Ca^2+^ (Ca_v_) channels [14], Trp channels [11] and store-operated Orai channels [15]. These channels are essential components in a multitude of signalling pathways and are also required for refilling of intracellular Ca^2+^ stores following intracellular Ca^2+^ release. Mechanisms of Ca^2+^ influx and demand for it are likely to differ between life cycle stages of many parasites, due to the differential ionic conditions of their respective environments. The intracellular location of many protozoan parasites during part of their life cycles [16], where Ca^2+^ concentrations in the mammalian host cell are maintained at low levels, suggests that novel Ca^2+^ entry pathways may be essential for parasite survival. In the extracellular trematode parasite *S. mansoni*, Ca^2+^ influx channels are important for muscle contraction and viability [17]. However, apart from the cloning of three *S. mansoni* Ca_v_ channels [18] and a report of Trp channels in various parasites [19], the identities of plasma membrane Ca^2+^ influx channels in pathogenic parasites remain largely unknown.

Pharmacological or genetic modulation of Ca^2+^ channel activity in the plasma membrane or intracellular organelles has profound effects on cell function and survival in many organisms. This suggests that the channels underlying Ca^2+^ signals in parasites might represent novel drug targets. Recent advances in genomics have led to whole-genome sequencing of several parasites that are pathogenic to humans (**Table 1**). In this study we examined the genomes of pathogenic parasitic protozoa, and that of *S. mansoni*, for the presence of genes that might encode Ca^2+^ channels in either the plasma membrane or intracellular organelles. We show that genes encoding homologues of mammalian intracellular Ca^2+^ channels and plasma membrane Ca^2+^ influx channels exist in many of these parasites. Some currently used anti-parasitic agents alter Ca^2+^ homeostasis in parasites, and some are also direct modulators of mammalian Ca^2+^ channels (see Discussion). This suggests that the parasite Ca^2+^ channel homologues described in this study might be the targets for some anti-parasitic drugs. Sequence divergence of parasite channels from their mammalian counterparts in regions that are important for channel activation, ion conduction or drug binding may result in distinct pharmacological profiles. These parasite channels may therefore represent attractive novel targets for rationally designed anti-parasitic therapies.

## Results

### Parasite homologues of IP_3_R and RyR pores

**Figure 1:**
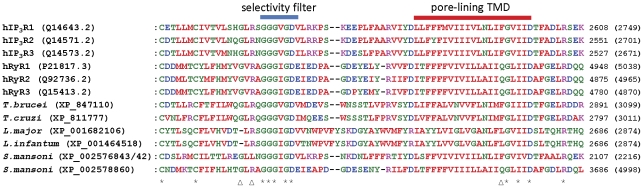
Alignment of parasite putative IP_3_Rs/RyRs with the pore regions of mammalian IP_3_Rs/RyRs. Alignments of the pore region of human IP_3_Rs and RyRs with putative homologues from *T. brucei, T. cruzi, L. infantum,* and *S. mansoni*. ClustalW2 physiochemical residue colours are shown, and protein accession numbers are shown in parentheses after the species name. Numbers in parentheses to the right of the alignments indicate the total number of residues in the protein. Asterisks below the alignment indicate absolute residue conservation in all homologues. Triangles below the alignment indicate the position of residues discussed in the text (G4891, R4893 and Q4934 of hRyR1). The blue bar above the alignment indicates the selectivity filter region, while the red bar indicates the pore-forming transmembrane domain (TMD). The *S. mansoni* protein labelled XP_002576843/42 is formed by concatenated XP_002576843 and XP_002576842 predicted proteins (whose genomic loci are adjacent). The entire concatamer was confirmed as a single intact protein by its identity with the predicted protein: 29191.m000804.twinscan2 (Wellcome Trust Sanger Institute, UK).

Mammalian IP_3_Rs and RyRs show a high degree of similarity, particularly in the pore region responsible for ion conduction. BLAST analysis of the conserved pore-region sequences of mammalian IP_3_Rs and RyRs against the whole-genome sequences of pathogenic parasites identified several predicted protein products displaying significant similarity in this pore region (**Figure 1** **and** **Table 2**). These homologues were found in the kinetoplastid *Leishmania* and *Trypanosoma* parasites, as well as in *S. mansoni*. In contrast to the multiple isoforms of IP_3_R and RyR found in vertebrates [7], [20], each species of *Trypanosoma* and *Leishmania* parasite possessed only a single homologue. In contrast, *S. mansoni* possessed two homologues: one similar to mammalian IP_3_Rs (XP_002576843/42 in **Figure 1**), which showed 34% identity with human IP_3_R3 (hIP_3_R3) and 31% identity with human RyR1 (hRyR1) in the full-length proteins; and one more similar to mammalian RyRs (XP_002578860), which showed 45% identity with hRyR2 and 32% identity with hIP_3_R1 (**Figure 1**). No IP_3_R/RyR homologues (of either pore or full-length sequences) were found in the genomes of any of the apicomplexan parasites, or in the protozoan parasites *E. histolytica, G. intestinalis* and *T. vaginalis*.

**Table 2:** Identity of Ca^2+^ channel homologues in pathogenic parasites.

Parasite	IP_3_Rs/RyRs	TPCs	Trp channels	Ca_v_ channels
*P. falciparum*	NF	NF	XP_001349872 (TrpML/TrpP) (14)^a^	NF
*P. knowlesi*	NF	NF	XP_002262030 (TrpML/TrpP) (14)^a^	NF
*P. vivax*	NF	NF	XP_001617062 (TrpML/TrpP) (12)^a^	NF
*T. gondii*	NF	XP_002364352 (15)	XP_002367104 (TrpP) (14) XP_002364302 (TrpP) (19)	XP_002370025 (6) XP_002367758 (2) XP_002367759 (11) XP_002368840 (12)
*C. hominis*	NF	NF	XP_668155 (TrpC/TpP/TrpV) (6) XP_667205 (TrpP) (13)	NF
*C. muris*	NF	NF	XP_002140701 (TrpC/TrpP) (8)XP_002139610 (TrpP) (17)	NF
*C. parvum*	NF	NF	XP_627518 (TrpC/TrpP/TrpV) (8) XP_627866 (TrpP) (13)	NF
*B. bovis*	NF	NF	NF	XP_001611701 (6)
*L. major*	XP_001682106 (10)	NF	XP_001681002 (TrpML) (6) XP_001684103 (TrpML/TrpP) (4)	XP_001682403 (21) XP_001686138 (24)
*L. infantum*	XP_001464518 (8)	NF	XP_001463306 (TrpML) (7) XP_001470439 (TrpML/TrpP) (7)	XP_001464813 (19) XP_001468437 (22)
*L. braziliensis*	NF	NF	NF	XP_001564333 (22) XP_001563925 (10)
*T. brucei*	XP_847110 (12)	NF	XP_845719 (TrpML/TrpP) (6)XP_846922 (TrpC/TrpP/TrpML) (6)	XP_822541 (22)
*T. cruzi*	XP_811777 (10)	NF	XP_804976 (TrpML/TrpP) (7)XP_804854 (TrpML/TrpP) (5)XP_816280 (TrpML/TrpP) (6)XP_807515 (TrpML/TrpP) (6)	XP_819699 (24)
*T. vaginalis*	NF	NF	XP_001325681 (TrpML/TrpP) (6) XP_001298159 (TrpML) (6) XP_001296819 (TrpV) (6)	NF
*S. mansoni*	XP_002576843/42 (IP_3_R) (4)XP_002578860 (RyR) (6)	XP_002578810 (4)	XP_002579262 (TrpML) (7) XP_002575977(TrpM) (7) XP_002570040 (TrpM) (9) XP_002579093(TrpM) (4) XP_002571459(TrpM) (4) XP_002573069(TrpM) (6) XP_002578456(TrpM/TrpC) (5)XP_002578457 (TrpM) (2) XP_002582108 (TrpM/TrpC/TrpV) (6) XP_002578454 (TrpM) (2) XP_002577419 (TrpP) (8) XP_002579176 (TrpP) (6) XP_002579107 (TrpP) (16) XP_002572123 (TrpA) (4) XP_002576849 (TrpC) (7) XP_002576118 (TrpC) (8) XP_002579731 (TrpC) (8) XP_002578785 (TrpC) (6) XP_002570832 (TrpC) (2)	XP_002578175 (20) XP_002575006 (18)AAK84311 (Ca_v_2A) (24) AAK84312 (Ca_v_1) (22) AAK84313 (Ca_v_2B) (20) XP_002571932 (18)^b^

Protein accession numbers for homologues are shown. Trp channel homologues were identified by homology with the transmembrane region of at least one class of mammalian Trp channel. Trp channels were then annotated according to the classes of mammalian Trp channel to which the full-length protein showed the greatest sequence similarity in BLASTP searches of the human genome (shown in parentheses for Trp channel homologues). Ca_v_ homologues include both human Ca_v_ channel and *S. cerevisiae* Cch1 homologues. The *S. mansoni* protein labelled XP_002576843/42 is formed by concatenated XP_002576843 and XP_002576842 predicted proteins (whose genomic loci are adjacent). The entire concatamer was confirmed as a single intact protein by its identity with the predicted protein: 29191.m000804.twinscan2 (Wellcome Trust Sanger Institute, UK). The number of predicted transmembrane domains (TMDs) present in homologues is indicated in parentheses. In addition to the Ca^2+^ channel homologues shown, a homologue of the store-operated Ca^2+^ influx channel Orai1 was found in *S. mansoni*: XP_002578837 (with shorter splice variant XP_002578838). Homologues of the very recently identified mitochondrial Ca^2+^ uniporter [124], [125] were also found in *Leishmania and Trypanosoma spp*. (eg. XP_822290 of *T. brucei*), as well as *S. mansoni* (XP_002569737), but were absent from the other parasites examined (*data not shown*). PM denotes plasma membrane and NF denotes no homologues found.

a Identified by homology with *T. gondii* protein XP_002367104;

b Possible Ca_v_2B fragment, incomplete sequence available.

### Structures of parasite IP_3_R and RyR homologues

**Figure 2:**
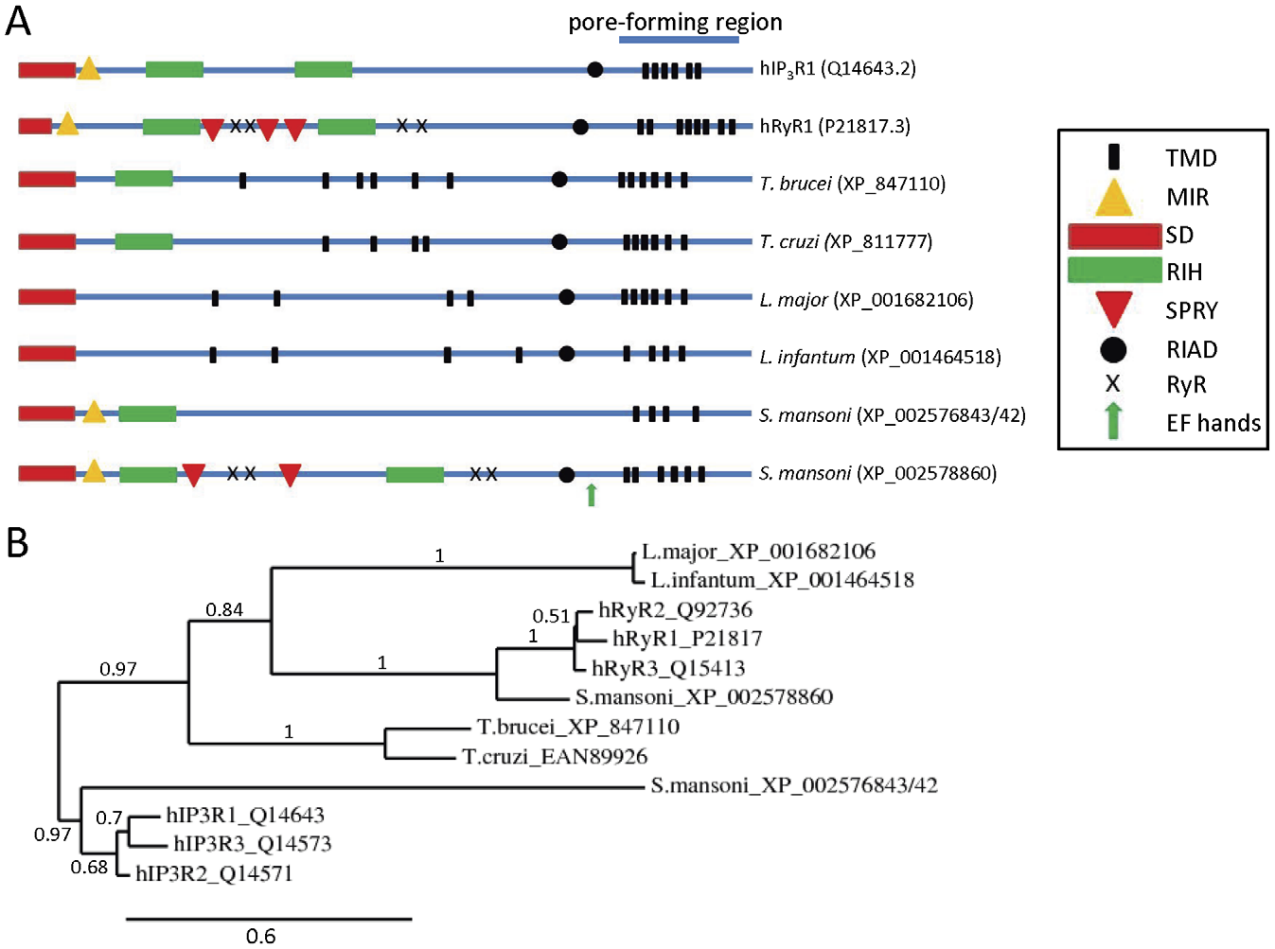
Comparison of full-length sequences of human and parasite IP_3_R/RyR homologues. (A) Schematic showing location of putative transmembrane domains (TMDs) and conserved domains identified using the Conserved Domains Database (NCBI), including: MIR (mannosyltransferase, IP_3_R and RyR) domains (pfam02815), RIH (RyR and IP_3_R homology) domains (pfam01365), RIH-associated domains (RIAD) (pfam08454), EF hands, suppressor-domain-like domains (SD) (pfam08709), RyR (ryanodine receptor) domains (pfam02026), and SPRY (SPla and the ryanodine receptor) domains (pfam00622). Putative pore-forming regions (pfam00520) are also indicated. (B) Phylogram showing relationships between full-length sequences of human and parasite IP_3_R/RyR homologues *(see Methods: based on 226 high confidence positions from a multiple sequence alignment; gamma shape parameter 1.755; proportion of invariant sites 0)*. Branch length scale bar and branch support values are shown.

Although the *Leishmania, Trypanosoma and S. mansoni* parasite proteins show a high degree of sequence similarity to IP_3_Rs/RyRs in the pore region, it was necessary to examine similarity in other regions before establishing them as potential functional correlates. Defining characteristics of vertebrate IP_3_Rs and RyRs include their large size (∼5000 residues for RyRs and ∼2700 residues for IP_3_Rs) and the presence of: multiple transmembrane domains near their C-terminal ends, conserved N-terminal MIR (mannosyltransferase, IP_3_R and RyR) domains, conserved RIH (internal RyR and IP_3_R homology) sequences with homology to the IP_3_-binding core of IP_3_Rs [21], conserved “RIH-associated domains” preceding their C-terminal transmembrane regions, and conserved N-terminal domains with homology to the suppressor domain of IP_3_Rs [22] (**Figure 2A**). Mammalian RyRs are also distinguished by the presence of multiple copies of a repeat termed an SPRY (SPla and RyR) domain and multiple copies of a repeat termed a RyR domain, both of which have unknown functions.

The parasite IP_3_R/RyR pore homologues are large proteins (2216-4998 residues) (**Figure 1**). The *S. mansoni* IP_3_R-like and RyR-like homologues are 2216 and 4998 residues in length, and phylogenetic analyses are consistent with these proteins representing IP_3_R and RyR homologues respectively (**Figure 2B**). Significantly, most homologues have predicted N-terminal RIH domains (**Figure 2A**), and searches of parasite genomes using the N-terminal IP_3_-binding domain sequence of mammalian IP_3_Rs (residues 224–604 of rat IP_3_R1) resulted in alignment with the same *Leishmania, Trypanosoma and S. mansoni* proteins found in the pore homology search. However, alignments suggested that like RyRs, the parasite homologues lack many of the ten basic residues known to be important for high-affinity binding of IP_3_ to mouse IP_3_R1 [23] (*data not shown*). The *S. mansoni* protein (XP_002576843/42), which has the greatest sequence similarity to full-length human IP_3_Rs, also has the highest conservation of these basic residues (5 out of 10) (*data not shown*). All parasite homologues are predicted to contain multiple putative transmembrane domains near their C-termini, and to contain N-terminal domains with similarity to the suppressor domain of mammalian IP_3_Rs and the analogous A-domain of RyRs [22] (**Figure 2A**). In addition, most homologues have N-terminal RIH-associated sequences (**Figure 2A**). Both *S. mansoni* homologues have N-terminal MIR domains, while the *S. mansoni* RyR homologue, like mammalian RyRs, has four copies of a RyR motif and multiple copies of an SPRY domain (**Figure 2A**). Several consensus Ca^2+^-binding EF-hands are also present in the C-terminus of the *S. mansoni* RyR homologue (**Figure 2A**), suggesting that this channel, like mammalian IP_3_Rs and RyRs, might be regulated by cytosolic Ca^2+^. Taken together, these sequence similarities suggest that these parasite channels are structural and functional analogues of mammalian IP_3_Rs and RyRs.

The close similarity of the pore regions of parasite channels to those of mammalian IP_3_Rs/RyRs, including the conserved GGGXGD selectivity filter motif (**Figure 1**) suggests that these parasite channels are likely to form cation channels with high single-channel conductance and permeability to Ca^2+^. Despite this significant similarity, parasite homologues show some potentially important sequence divergence from human IP_3_Rs/RyRs in the pore region (**Figure 1**). For example, both *Leishmania* homologues and the *S. mansoni* IP_3_R-like homologue differ from human IP_3_Rs/RyRs near the selectivity filter region, at positions analogous to hRyR1 residues G4891 and R4893 that are known to affect ryanodine binding [24]. Also, all parasite homologues (except the *S. mansoni* RyR homologue) differ from human RyRs in the pore-lining transmembrane domain, at the position analogous to Q4934 of hRyR1, which is a determinant of ryanodine binding [25]. Some pathogenic parasites therefore possess homologues of both IP_3_Rs and RyRs in which key functional domains are well enough conserved to suggest that these proteins might function as intracellular Ca^2+^ channels. However, the sensitivity of these parasite channels to drugs may differ sufficiently from those of their host to perhaps allow their selective targeting by drugs.

### TPC homologues

**Figure 3:**
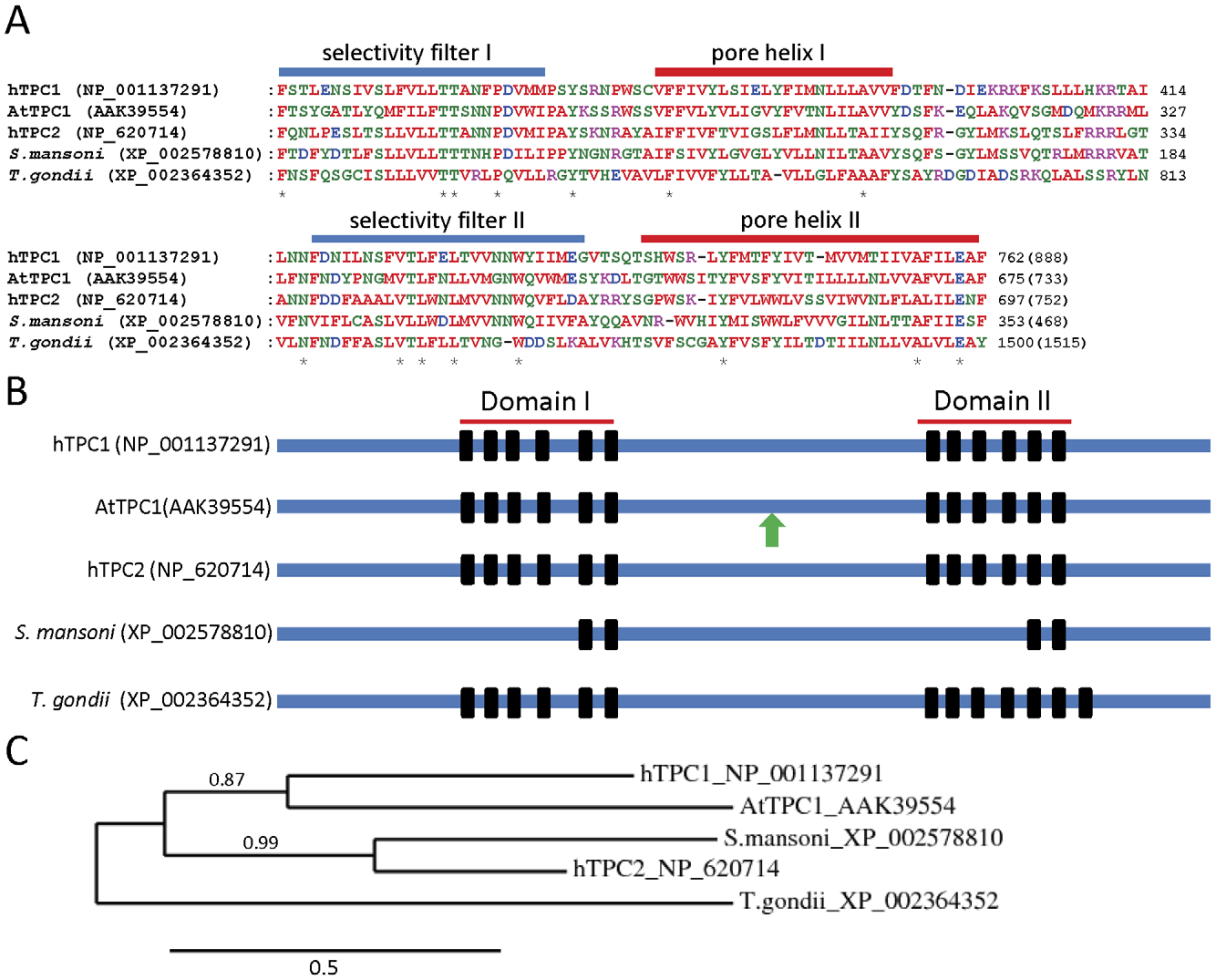
Comparison of human, plant and parasite TPC homologues. (A) Alignments of pore domains I (upper panel) and II (lower panel) of human, plant (AtTPC1 is *Arabidopsis thaliana* TPC1) and parasite TPC homologues. Asterisks below the alignment indicate absolute residue conservation in all homologues. (B) Schematic showing the location of transmembrane domains (black bars) within the full-length sequences of human, plant and parasite TPC homologues. The green arrow signifies an EF-hand repeat in AtTPC1. (C) Phylogram showing the relationship between full-length sequences of human, plant and parasite TPC homologues *(see Methods: based on 220 high confidence positions from a multiple sequence alignment; gamma shape parameter 4.195; proportion of invariant sites 0.021)*. Branch length scale bar and branch support values are shown.

We searched next for parasite homologues of mammalian TPCs, which mediate release of Ca^2+^ from acidic organelles in mammalian cells. A defining characteristic of both mammalian and plant TPCs is the presence of two independent pore-forming domains in series, each consisting of multiple transmembrane domains [8], [9], [26], [27]. We therefore searched parasite genomes, using the full-length sequences of human TPC1 and TPC2, for homologues that contained at least four transmembrane domains in two distinct regions. Reciprocal BLAST searches were then carried out with sequences of the identified parasite proteins, to confirm specific homology with mammalian TPCs. This approach identified TPC homologues in only *S. mansoni* and *T. gondii* (**Figures 3A and 3B**). These parasite homologues show substantial similarity to mammalian TPCs in the pore regions responsible for ion conduction (**Figure 3A**) suggesting that they, like their mammalian counterparts, may act as Ca^2+^-permeable channels. The *S. mansoni* homologue shows most similarity to human TPC2, while the *T. gondii* homologue is more distantly related to mammalian TPCs (**Figure 3C**). Interestingly, the *S. mansoni* TPC homologue has only two predicted transmembrane segments in each of its pore domains, compared to the six found in each domain of mammalian TPCs [8], [9], [26], [27]. The sequences of plant TPC (*Arabidopsis thaliana*, AtTPC1) as well as the *S. mansoni* and *T. gondii* TPC homologues themselves were then used to search for additional parasite homologues, but none were identified. The parasite TPC homologues identified here lack the EF-hand repeat found in plant AtTPC1 channels (**Figure 3B**), suggesting that like mammalian TPCs, parasite TPC homologues may be Ca^2+^-insensitive.

### Intracellular Trp channel homologues

**Figure 4:**
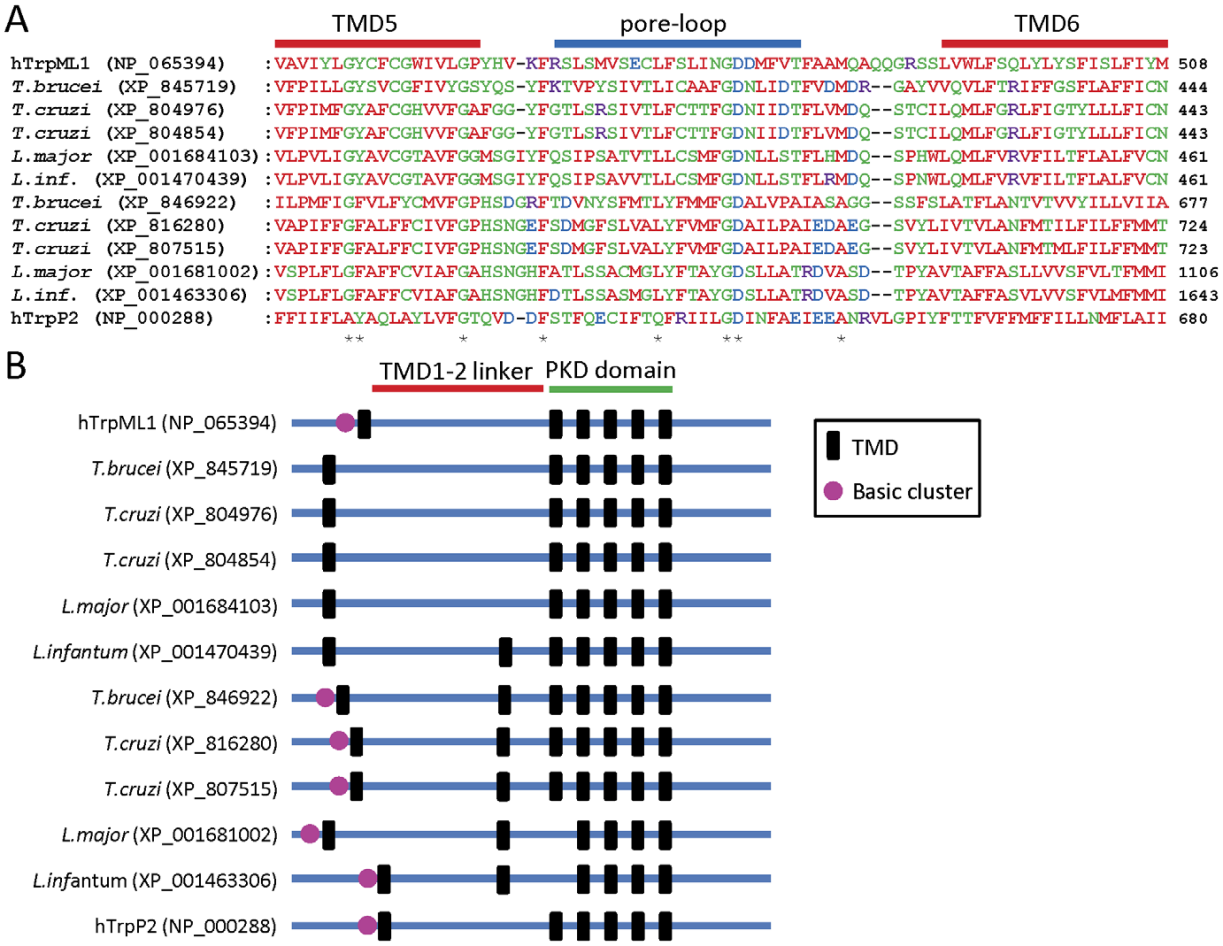
Trp channel homologues in kinetoplastid parasites. (A) Alignment of the pore regions of human TrpML and TrpP2 channel subunits with putative homologues from kinetoplastid parasites. Sequences of the putative pore loops as well as part of the TMD5 and TMD6 regions are shown. Numbers in parentheses to the right of the alignments indicate the total number of residues in the protein. Asterisks below the alignment indicate residues that are absolutely conserved between parasite proteins and either hTrpML1 or hTrpP2. *L. infantum* is shown abbreviated to *L. inf.* (B) Schematic showing the location of predicted TMDs (black bars) within the full-length sequences of human TrpML1 and TrpP2 channel subunits, as well as parasite homologues. The conserved PKD polycystin domain (pfam08016; identified using the Conserved Domains Database, NCBI) is indicated, as well as the long TMD1-TMD2 linker characteristic of mammalian TrpML and TrpP2 channel subunits. The presence of an N-terminal cluster of basic residues preceding TMD1 is indicated by a purple circle.

In addition to IP_3_Rs, RyRs and TPCs, several subtypes of mammalian Trp channel are located within the membranes of intracellular organelles and mediate Ca^2+^ release in mammalian cells. These include TrpM, TrpML, and TrpP2 channels [10], [11], [28]. We therefore searched parasite genomes for homologues of these Trp channels, using the full-length and N-terminally truncated (to remove common ankyrin domains) sequences of human isoforms, followed by further searches using the sequences of identified homologues from *T. gondii*. We identified many parasite homologues of these Trp channels (**Table 2**). Putative Trp channel homologues in *Plasmodium spp*. were only identified by homology with a *T. gondii* Trp channel homologue, and show only weak similarity to mammalian Trp channels. Overall, these results are consistent with a recent report of Trp channels in various parasites [19], although we describe here additional putative homologues in *Plasmodium spp*., *T. gondii*, *T. vaginalis*, *Cryptosporidium spp*., *Trypanosoma spp*. and *S. mansoni* (**Table 2**). Definitive categorization of homologues as subtypes of Trp channel was difficult, as multiple homologies existed between these proteins. Homologues were therefore categorized by reference to the human Trp channels to which they showed the greatest sequence similarity (**Table 2**). TrpML and TrpP homologues were found in most parasites examined, while TrpM channel homologues were found only in *S. mansoni* (**Table 2**). We were most interested in the TrpML/TrpP homologues found in kinetoplastid parasites, which show considerable similarity to human TrpML and TrpP2 channel subunits in the putative pore region (**Figure 4A**). They also have conserved predicted polycystin (PKD) domains and long TMD1-TMD2 linkers that are characteristic of TrpML and TrpP channel subunits (**Figure 4B**). In addition, several of these parasite proteins have a cluster of basic residues preceding their TMD1 regions, similar to the cluster responsible for binding phosphatidylinositol 3,5-bisphosphate (PI(3,5)P_2_) in mouse TrpML1 [29] (**Figure 4B**). The Trp channel homologue Yvc1p (TrpY1) is responsible for mediating Ca^2+^ release from vacuolar stores of *Saccharomyces cerevisiae* [30], [31]. We therefore searched parasite genomes for homologues of full-length Yvc1p, but none were found.

### Plasma membrane Trp channel homologues

Ca^2+^-permeable Trp channels located in the plasma membrane of mammalian cells include TrpC, TrpA, TrpV and TrpP subtypes [11], [28]. TrpC channels may contribute to store-operated Ca^2+^ entry in many cells [32], TrpA and TrpV channels respond to external chemical and thermal stimuli [11], and TrpP2 channels at the plasma membrane respond to stimuli including mechanical stress and receptor activation [33]. We searched parasite genomes for homologues of these Trp channels, using the full-length and N-terminally truncated (again, to remove ankyrin domain hits) sequences of human isoforms, and identified several parasite homologues (**Table 2**). The protozoan parasite genomes examined were found to lack homologues of TrpA and TrpC channels, and only *T. vaginalis* had a homologue of TrpV channels. In contrast, *S. mansoni* was found to contain TrpA and TrpC channel homologues (**Table 2**). The presence of TrpP homologues was discussed earlier, in the context of intracellular Trp channels. The Trp channel homologues identified are consistent with a recent report of Trp channels in parasites [19], with additional homologues found to exist in *S. mansoni* (XP_002576118) and *T. vaginalis* (XP_001296819) in the current study (**Table 2**).

#### Orai and STIM homologues

Store-operated Ca^2+^ entry (SOCE) is an almost ubiquitous feature of mammalian cells, where plasma membrane channels composed of Orai subunits mediate Ca^2+^ influx in response to emptying of intracellular Ca^2+^ stores [34]-[37]. STIM subunits act as the sensors of ER Ca^2+^ depletion and are necessary for activation of Orai channels [38]–[40]. However, apart from a single study suggesting the presence of SOCE in *Plasmodium falciparum* [41], the existence of SOCE in parasites is largely unexplored, and whether Orai and STIM homologues exist in parasites is not known.

**Figure 5:**
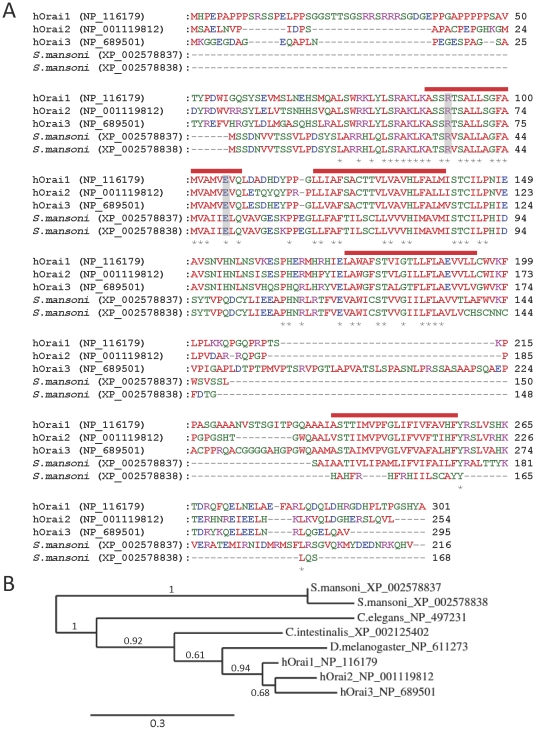
Comparison of human and *S. mansoni* Orai homologues. (A) Alignments of human Orai subunits with predicted homologues from *S. mansoni* are shown (XP_002578838 is a shorter alternatively spliced variant of XP_002578837). Residues within the putative pore that are crucial for hOrai1 function (R91 and E106) are highlighted by grey bars. Predicted transmembrane regions of hOrai1 are indicated by red bars above the alignment, and asterisks below the alignment denote sequence identity amongst all isoforms. (B) Phylogram showing the relationship between human and *S. mansoni* Orai homologues as well as those of the model invertebrates *C. elegans, D. melanogaster and C. intestinalis (see Methods: based on 141 high confidence positions from a multiple sequence alignment; gamma shape parameter 1.541; proportion of invariant sites 0.156)*. Branch length scale bar and branch support values are shown.

Parasite genomes were searched for homologues of these proteins, using full-length sequences of human Orai1 and STIM1 proteins. All pathogenic apicomplexan parasites examined were found to lack both Orai and STIM homologues. In contrast, the *S. mansoni* genome contains a gene encoding an Orai1 homologue (XP_002578837), with an alternatively spliced variant (XP_002578838) (**Figures 5A and 5B**), and a homologue of STIM1 (XP_002581211). The Orai homologue shows pronounced identity with human isoforms in the pore region (**Figure 5A**), including residues R91 and E106 of human Orai1, which are critical for pore function [34], [42], [43]. The occurrence of both Orai and STIM homologues in *S. mansoni* strongly suggests that a SOCE process similar to that found in mammalian cells exists in this parasite.

### Ca_v_ channels

**Figure 6:**
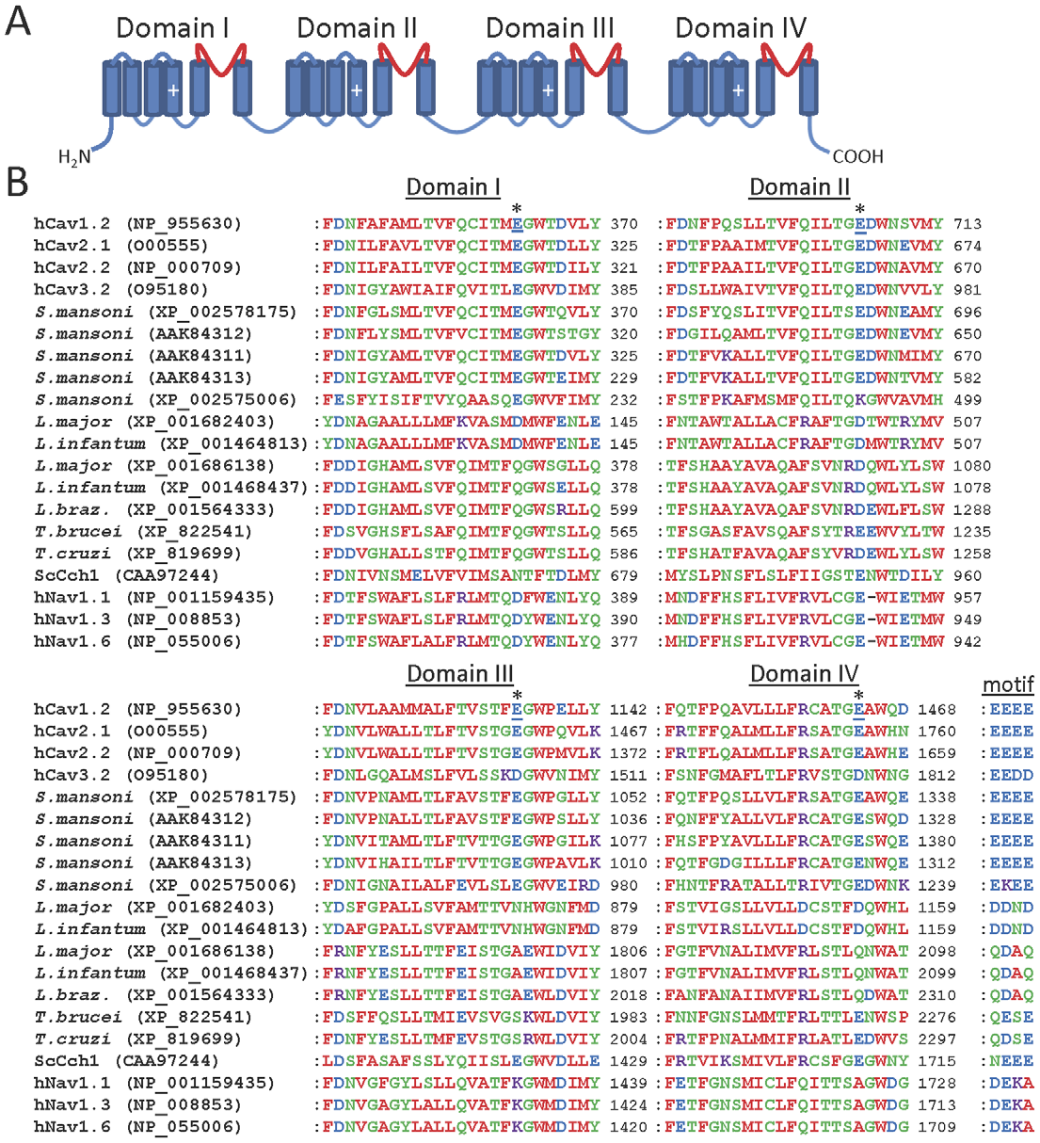
Parasite Ca_v_ channel homologues show similarity to human Ca_v_ channels in the pore region. (A) Schematic showing the four-domain structure of human Ca_v_ channels, with the pore loop of each domain shown in red. Cylinders indicate TMDs, and plus signs indicate the charged voltage sensor regions. (B) Multiple sequence alignments of the pore domains of human Ca_v_ channels with parasite Ca_v_ channel homologues. Only those parasite homologues containing four putative domains are shown. Sequences of human Ca_v_1.2 (L-type), Ca_v_2.1 (P/Q-type), Ca_v_2.2 (N-type) and Ca_v_3.2 (T-type) channels, as well as the *S. cerevisiae* Cch1 Ca^2+^ channel (ScCch1), are shown. *L. braz* denotes *L. braziliensis*. Selected human Na_v_ isoforms are included, to allow comparison with the related Ca_v_ channels. The locations of acidic residues (underlined) forming an acidic ring motif in human Ca_v_1.2 channels are indicated by asterisks. The overall motif formed by all four domains at this locus is indicated to the right of the Domain IV alignment for each channel homologue.

**Figure 7:**
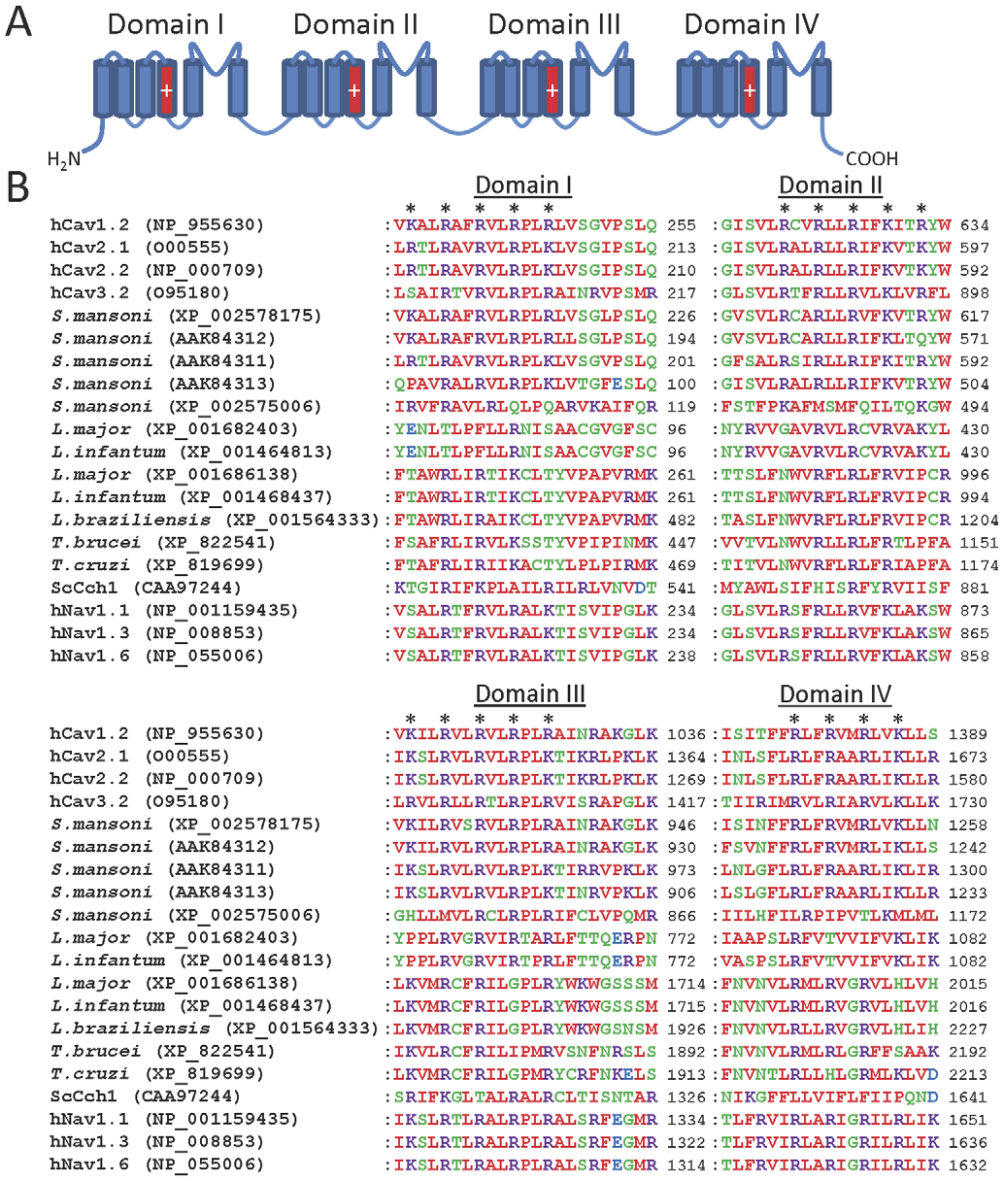
Parasite Ca_v_ channel homologues show similarity to human Ca_v_ channels in the voltage sensor region. (A) Schematic showing the four-domain structure of human Ca_v_ channels, with the voltage sensors of each domain shown in red. (B) Multiple sequence alignments of the voltage sensor domains of human Ca_v_ channels with parasite Ca_v_ channel homologues. Only those parasite homologues containing four putative domains are shown. Asterisks indicate the position of basic residues which form the voltage sensor in each domain of human Ca_v_1.2 channels.

Many subytpes of plasma membrane Ca_v_ channel exist that are crucial for voltage-dependent Ca^2+^ influx in a wide variety of mammalian cells [14]. Human Ca_v_ channel sequences and the sequence of the Cch1 Ca_v_ channel homologue from *S. cerevisiae* [44], [45] were used to search for homologues in parasites. *S. mansoni* was found to possess genes encoding several Ca_v_ channel homologues. In addition to the previously described and cloned Ca_v_1, Ca_v_2A and Ca_v_2B channels [18], we identified two novel putative Ca^2+^ channels (XP_002578175 and XP_002575006) (**Table 2**). A partial sequence of another potentially novel Ca_v_ homologue was also identified (XP_002571932), which shows pronounced (but not perfect) similarity with Ca_v_2B, but insufficient sequence was available to conclusively categorize this protein. Ca_v_ channel homologues were also found in all other parasites examined except *Plasmodium spp*., *Cryptosporidium spp., T. vaginalis, G. intestinalis and E. histolytica* (**Table 2** and **Figure 6**). We also searched parasite genomes for homologues of the CatSper channels (relatives of Ca_v_, TPC and Trp channels) that are responsible for Ca^2+^ entry into mammalian spermatozoa [46]. All CatSper homologues found were identical to the Ca_v_ homologues identified above. Some of the Ca_v_ channel homologues identified in parasites are predicted to possess 18–24 transmembrane domains (**Table 2**), consistent with a four-domain structure formed from a single subunit, similar to the organization of mammalian Ca_v_ channels (**Figure 6A**) [14]. In contrast, some of the parasite Ca_v_ channel homologues, such as those found in *T. gondii* and *B. bovis*, possess only 2–6 predicted transmembrane domains (**Table 2**), suggesting that these may be a novel class of Ca_v_ channel homologue, formed by tetramerization of four individual subunits. Like mammalian Ca_v_ channels and Cch1, most parasite Ca_v_ channel homologues have acidic residues within their putative pore loops (**Figure 6B**). In mammalian Ca_v_ channels these residues form a negatively charged ring in the intact tetrameric channel, which facilitates selectivity for Ca^2+^ [14]. Fewer acidic residues are present at the analogous positions in some homologues from *Leishmania spp*. and *Trypanosoma spp*., although several acidic residues are present at other positions within the putative pore loops of these proteins (**Figure 6B**). Voltage-gated sodium (Na_v_) channels are thought to have evolved from Ca_v_ channels and share a high degree of sequence similarity with them [47]. It is possible therefore that some of the Ca_v_ homologues identified here may be selective for Na^+^ rather than Ca^2+^ (or they may be non-selective), although experimental testing will be required to determine this. However, all parasite homologues identified lacked the characteristic DEKA selectivity filter motif of mammalian Na_v_ channels, which is necessary for Na^+^ selectivity [48] (**Figure 6B**). Many of the parasite Ca_v_ channel homologues possess several regularly spaced basic residues in the TMD4 region of each domain (**Figure 7A and 7B**), suggesting functional equivalence of these regions to the voltage sensors of mammalian Ca_v_ channels [**14**].

### Other Ca^2+^-permeable channels

Given the relative sparsity of Ca^2+^ channel homologues in the protozoan parasites studied, we also searched their genomes for genes encoding homologues of other mammalian Ca^2+^-permeable channels. Cyclic nucleotide-gated (CNG) cation channels can mediate Ca^2+^ influx in mammalian cells [49]. We searched parasite genomes for CNG homologues, using the full-length and transmembrane region-only (to remove common cyclic nucleotide-binding domains) sequences of human CNGA1 and CNGA2. Only hits containing multiple transmembrane domains and which showed similarity in the pore and ligand-binding regions were acknowledged. No homologues were found in any of the protozoan parasites examined, except for several homologues in *T. vaginalis* which all contain the pore motif GYGD reminiscent of potassium channels [50]. This suggests that they may be K^+^-selective channels rather than Ca^2+^ channels, although this requires experimental testing.

We also searched protozoan genomes for genes encoding homologues of potentially Ca^2+^-permeable mammalian NMDA receptors, kainate receptors, AMPA receptors, pannexins, P2X4 purinergic receptors and nicotinic acetylcholine receptors. No convincing homologues (*ie*. showing multiple transmembrane domains, pore homology, and reciprocal BLAST output) of these channels were found in any of the protozoan parasite genomes examined. In contrast to protozoan parasites, *S. mansoni* is known to have homologues of some of these receptors, such as nicotinic acetylcholine receptors [51], [52] and P2X receptors [53], but analysis of these likely non-selective cation channel homologues in this organism was not extended further.

## Discussion

Ca^2+^ channels are critical for many of the most fundamental cellular processes and pharmacological modulation of these channels can lead to marked changes in cell growth and viability. These channels therefore represent attractive drug targets for treatment of infectious disease. Complex Ca^2+^ signalling processes exist in parasites and Ca^2+^-handling machinery including Ca^2+^-ATPases is present in these organisms [2], [3]. We have shown that genes encoding proteins homologous to mammalian intracellular Ca^2+^ channels and Ca^2+^ influx channels are present in many of the most clinically relevant pathogenic parasites (**Table 2**). Many of these putative channels are not yet annotated in available pathogen databases, such as ToxoDB and TriTrypDB (http://eupathdb.org/eupathdb) [54]. The presence of these Ca^2+^ channel homologues, along with the occurrence of physiological Ca^2+^ signals in parasites, suggests that they participate in Ca^2+^ signalling within these cells. Our analysis has attempted to distinguish intracellular from plasma membrane Ca^2+^ channels because the distribution of Ca^2+^ channels profoundly affects their regulation, the amount of Ca^2+^ to which they have access, and the subcellular organization of the Ca^2+^ signals they evoke [55]. It is however increasingly clear that many Ca^2+^ channels can function effectively in both intracellular organelles and the plasma membrane [55]. Some parasites, whose extracellular surface may, at different stages of their lifecycle, be exposed to typical extracellular Ca^2+^ concentrations or to the 10,000-fold lower Ca^2+^ concentration of their host cell's cytosol, may perhaps exploit this plasticity in targeting of Ca^2+^ channels even more extensively than mammalian cells. Despite the difficulty of unambiguously assigning Ca^2+^ channel homologues to specific membrane compartments on the basis of their primary sequence alone, subsequent sections consider the parasite homologues by reference to the distribution of their mammalian counterparts.

### Parasite intracellular Ca^2+^ channels

Pathogenic parasites contain a variety of intracellular Ca^2+^ stores including ER, mitochondria, glycosomes and acidocalcisomes, and biochemical signalling pathways analogous to those of mammalian cells have been shown to exist in parasites. Evidence exists for the presence of phospholipase C signalling pathways in parasites [56]–[58], which may sustain IP_3_-based effects on IP_3_R homologues. The intracellular messenger cADPR that activates mammalian RyRs has also been shown to be involved in parasite signalling pathways [59]–[60]. Which of these pathways might regulate the IP_3_R/RyR homologues shown in this study to exist in *Leishmania*, *Trypanosoma* and *S. mansoni* parasites remains to be tested. However, previous observations indicate that IP_3_ lacks Ca^2+^-releasing activity in *Trypanosoma* parasites [61]–[62]. In addition, parasite IP_3_R/RyR homologues, like mammalian RyRs, lack many of the basic residues required for high-affinity binding of IP_3_ in mouse IP_3_R1 [23]. These observations suggest that the IP_3_R/RyR homologues in these organisms might respond to a different intracellular messenger (such as cADPR), or that they might require specific conditions for activity in response to IP_3_.

Apicomplexan parasite genomes appeared to lack IP_3_R/RyR homologues, as reported by others [63]. Despite the absence of IP_3_R/RyR homologues in these parasites, IP_3_ has been reported to elicit Ca^2+^ release from intracellular stores of *Plasmodium chabaudi* [64] and *E. histolytica* [65], and Ca^2+^ release has been attributed to IP_3_R/RyR-like channels in *T. gondii* [66] and *Plasmodium berghei* [67]. These observations suggest that another type of IP_3_-sensitive intracellular Ca^2+^ channel exists in apicomplexan parasites. A similar situation is seen in *S. cerevisiae*, which lacks IP_3_R/RyR homologues, but has been reported to show IP_3_-induced Ca^2+^ release from vacuolar vesicles [68]. The Yvc1p (TrpY1) channel is responsible for mediating Ca^2+^ release from vacuolar stores of *S. cerevisiae* [30]–[31] and this channel is sensitive to PI(3,5)P_2_ [29], although whether this channel is also sensitive to IP_3_ is not yet known. Our searches found no homologues of Yvc1p among the genomes of pathogenic apicomplexa. However, we did identify apicomplexan homologues of the PI(3,5)P_2_-sensitive mammalian endolysosomal TrpML1 channel [29]. Whether mammalian TrpML channels are sensitive to IP_3_ has not been tested, so whether these homologues might mediate the IP_3_-sensitive Ca^2+^ release in apicomplexan parasites remains uncertain. The molecular determinants of IP_3_-evoked Ca^2+^ release in apicomplexan parasites therefore remain to be determined. The Trp, TPC, Cch1 and Ca_v_ channel homologues identified in this study (see below and **Table 2**) are also potential candidates for the role of novel IP_3_-sensitive intracellular Ca^2+^ channels in the pathogenic parasites that lack IP_3_R/RyR homologues.

Trp channels in mammalian cells can mediate Ca^2+^ release from intracellular stores in response to a wide variety of stimuli [10], [11]. In addition, the Trp channel homologue Yvc1p is the dominant Ca^2+^ release channel in *S. cerevisiae* [30], [31]. Trp channel homologues are therefore likely candidates for mediating intracellular Ca^2+^ release in parasites, consistent with previous identification of TrpML genes in *L. major* [69] and a recent report published during the course of this study which noted the presence of Trp channel homologues in a variety of parasites [19]. We identified a striking abundance of Trp channel homologues in parasites (**Table 2**), including many TrpML homologues, which in mammalian cells have been shown to play roles in signal transduction, ion homeostasis and membrane trafficking [29], [70]. TrpP homologues are also apparent in many parasites (**Table 2**). In mammalian cells these channels can mediate release of Ca^2+^ from ER stores and can be modulated by cytosolic Ca^2+^ via an EF-hand domain [10], [71], [72]. Whether these parasite Trp channels play similarly diverse roles in Ca^2+^ release, signal transduction and membrane trafficking remains an exciting avenue for future research.

The parasite acidocalcisome is similar in several ways to mammalian lysosomes from which TPCs [8], [9], TrpM [73] and TrpML [29], [70] channels mediate Ca^2+^ release in mammalian cells. Both organelles have a low luminal pH, high Ca^2+^ content and enzymatic activity [74]. TPCs in mammals and sea-urchins are regulated by intracellular NAADP [8], [9], plant TPCs may be regulated by Ca^2+^ [75], TrpML channels are modulated by PI(3,5)P_2_ [29] and TrpM2 channels are modulated primarily by adenosine diphosphoribose (ADPR), as well as cADPR and Ca^2+^ [73]. However, no second messenger has yet been shown to cause release of Ca^2+^ from acidocalcisomes [74]; neither NAADP nor IP_3_ releases Ca^2+^ from acidocalcisomes of sea urchin eggs [76]; and the effect of NAADP and ADPR on Ca^2+^ dynamics in parasites has not been studied. Interestingly, three of the TrpM homologues in *S. mansoni* (XP_002573069, XP_002571459 and XP_002578454) contain Nudix hydrolase domains, suggesting that they might be involved in signalling pathways utilizing nucleoside diphosphate derivatives such as ADPR or cADPR, as is the case with mammalian TrpM channels [77], [78]. Some TrpML homologues in kinetoplastid parasites contain a cluster of basic residues before their TMD1 regions. A similar cluster allows binding of PI(3,5)P_2_ to mammalian TrpML channels and subsequent channel activation [29], suggesting that the parasite homologues may also form phosphoinositide-sensitive channels. Whether the parasite homologues of mammalian TPCs or other intracellular Ca^2+^ channels identified in this study release Ca^2+^ from acidocalcisomes or other organelles in response to NAADP, ADPR, cADPR, PI(3,5)P_2_, IP_3_, Ca^2+^, or other intracellular messengers therefore remains to be determined.

### Parasite Ca^2+^ influx channels

Ca^2+^ influx mediates physiological signalling pathways in parasites [79]–[84] and allows refilling of intracellular Ca^2+^ stores following intracellular Ca^2+^ release. The importance of Ca^2+^ influx pathways for cell survival in general has been demonstrated in *S. cerevisiae*, where lack of the plasma membrane Cch1 Ca^2+^ channel impairs high-affinity Ca^2+^ uptake, and leads to cell death in conditions of low Ca^2+^ concentration or when Ca^2+^ influx is required [44], [45]. Recently it has likewise been shown that in *Leishmania* parasites, Ca^2+^ influx is necessary for thermotolerance [84]. Many protozoan parasites exist within mammalian cells for part of their life cycle, where Ca^2+^ levels are maintained at low levels (typically <100 nM). These parasites therefore seem likely to require unique strategies in order to enhance Ca^2+^ uptake. Residence of some parasites within parasitophorous vacuoles or other membraneous compartments [16], [85] helps to circumvent this problem by surrounding the parasite with elevated Ca^2+^, but parasite plasma membrane Ca^2+^ channels or transporters with novel properties may also be essential, especially for those parasites directly exposed to the cytosol, such as *T. cruzi* [16], [86]. The Ca^2+^ channel homologues described in this study may differentially contribute to Ca^2+^ influx during different phases of parasite life cycles and may have novel properties that allow their function in ionic conditions that differ substantially from those experienced by their mammalian counterparts.

Ca_v_ channel homologues were found in many of the pathogenic parasites examined, suggesting that these proteins have a widespread function in parasites. The presence in these homologues of charged regions analogous to the voltage sensors of Ca_v_ channels suggests that they might be gated by transmembrane voltage, although this remains to be tested experimentally. Homologues of mammalian Ca^2+^-permeable TrpA, TrpC and TrpV subtypes capable of mediating plasma membrane Ca^2+^ influx were absent from most protozoan parasites. In contrast, TrpA and TrpC channel homologues were found in *S. mansoni*, although their exact subtype categorization was unclear. Since in addition to mediating intracellular Ca^2+^ release, TrpP channels are also capable of mediating Ca^2+^ influx in mammalian cells when located in the plasma membrane [33], [87], the “intracellular” Trp channel homologues identified in this study may also contribute to Ca^2+^ *influx* in parasites. Mammalian Trp channels are modulated by diverse stimuli [10], [11], [32] making them interesting candidates for the transduction of environmental stimuli into physiological responses in *S. mansoni*.

Homologues of the Orai channels which mediate SOCE in mammalian cells [34]–[37], were found to be absent from all pathogenic protozoa examined. In addition, the ER Ca^2+^-sensing STIM proteins responsible for activation of Orai proteins [38]–[40] were also absent from these parasites. This suggests that either these parasites lack SOCE, or that other proteins might fulfil the roles of both the pore-forming and Ca^2+^-sensing subunits of SOCE channels in these organisms. In mammalian cells, TrpC channels have also been shown to contribute to SOCE in a variety of cells [88], suggesting the possibility that Trp channel homologues (**Table 2**) might fulfil roles as SOCE channels in some of these protozoa. Interestingly, in contrast to the absence of Orai/STIM proteins in the protozoan parasites tested, *S. mansoni* has an alternatively spliced Orai1 homologue and a STIM1 homologue, suggesting that Orai/STIM-mediated SOCE exists in this parasite.

The expression and function of the putative Ca^2+^ channel homologues identified in this study will in future need to be measured experimentally in order to confirm their status as Ca^2+^ channels. Homologues of other mammalian non-selective cation channels may also be found in parasites and may contribute to Ca^2+^ signalling pathways in these organisms. In addition, some of the Ca^2+^ channel homologues identified in this study may play roles in flux of other ions such as Na^+^ or K^+^, perhaps in a lifecycle-dependent manner, given the substantial changes in ionic composition of the extracellular environment during different stages of the lifecycle in many parasites.

### Ca^2+^ channel homologues in parasites and their free-living relatives

Comparison of the genomes of the parasitic Apicomplexa examined here (*Plasmodium spp*., *T. gondii* and *Cryptosporidium spp*.) with their free-living ciliate relative *Paramecium* reveals differences in the complement of genes encoding Ca^2+^ channels. IP_3_R/RyR homologues are absent in the Apicomplexa examined, whereas they are present in *Paramecium* [89]. Likewise, Ca_v_ channels with a four-domain architecture similar to mammalian Ca_v_ channels are absent in the Apicomplexa (*T. gondii* has several more distantly related homologues, but these do not have a four-domain structure), whereas they are present in *Paramecium (data not shown)*. Whether the apparent absence of IP_3_R/RyR and Ca_v_ channels in Apicomplexa occurred as a result of the acquisition of a parasitic existence is unclear. In contrast, both the Apicomplexa examined here and *Paramecium* (*data not shown*) appear to have Trp channel homologues. *Paramecium* [90] and the apicomplexan parasite *P. falciparum* [41] have been reported to display SOCE, although the molecular basis is unknown. As with the Apicomplexa, our searches did not reveal any genes encoding convincing homologues of Orai1 in *Paramecium (data not shown)*, suggesting that lack of Orai homologues in Apicomplexa may not be a result of transition to parasitism. The channel-forming proteins underlying SOCE in these organisms remain to be identified.

A comparison of the flagellate parasites examined here (*Trypanosoma spp*. and *Leishmania spp*.) with their free-living choanoflagellate relative *Monosiga brevicollis* also reveals differences. Both *M. brevicollis* [91] and the kinetoplastid parasites examined here have homologues of IP_3_Rs, Ca_v_ and Trp channels, although *M. brevicollis* has a greater diversity of these channels [91]. However, while Orai and STIM homologues are present in *M. brevicollis* [91], they appear to be absent in the kinetoplastid parasites. Again, whether these differences arose due to the transition from a free-living to a parasitic existence is unclear.

### Relevance of parasite Ca^2+^ channels to therapy

A role for Ca^2+^ in the action of some anti-parasitic drugs has long been appreciated [92] and many anti-parasitic drugs that are known to affect mammalian Ca^2+^ channels have effects on Ca^2+^ signalling in parasites. For example, amiodarone [93], nimodipine [94] and several other 1,4-dihydropyridines [95], as well as a range of Ca^2+^ channel and calmodulin antagonists [96] may exert anti-parasitic effects via disruption of Ca^2+^ homeostasis in parasites. Anti-parasitic actions of other agents such as antimicrobial peptides [97], parasite-specific antibodies [98] and curcumin [99] also affect Ca^2+^ homeostasis in parasites. In addition, the widely used antimalarial artemisinin may affect Ca^2+^ homeostasis via inhibition of parasite sarco/endoplasmic reticulum Ca^2+^-ATPase (SERCA) [100], although this has recently been contested [101], [102] and other mechanisms for its action have been hypothesized [103]. Praziquantel [104], [105], chloroquine [106], [107], dantrolene [59], [108], and suramin [109], [110] are also currently used anti-parasitic drugs with complex and unclear mechanisms of action, that may involve modulation of Ca^2+^ signalling. We speculate that in addition to affecting their currently known targets, such as the plasmodial surface anion channel in the case of dantrolene [111] and Ca_v_β subunits in the case of praziquantel [112], these drugs may also alter the activity of the parasite Ca^2+^ channel homologues described in this study and hence perturb Ca^2+^ signalling pathways involved in parasite survival. In particular, we suggest that suramin, a known RyR agonist [113], [114] and dantrolene, a known RyR antagonist [115], [116], may affect the RyR/IP_3_R homologues shown in this study to exist in *Trypanosoma*, *Leishmania* and *S. mansoni* parasites. This represents a potentially novel mechanism of action for these clinically useful drugs.

The apparent scarcity of Ca^2+^ channels in parasites relative to the wide array of isoforms in mammalian cells suggests that Ca^2+^ signalling in parasites may rely on a less redundant repertoire of Ca^2+^ channels. This characteristic may increase the susceptibility of parasites to drugs that target these channels.

Although parasite Ca^2+^ channel homologues show significant similarity to their mammalian counterparts, sequence differences in functionally and pharmacologically important domains (eg. the ion-conducting pore region) exist, such as those shown to exist between human and parasite IP_3_R/RyR homologues (**Figure 1**). As pharmacological blockers of IP_3_Rs and RyRs such as xestospongin-C and ryanodine are thought to bind within the pore region [24], [25], [117], this sequence divergence suggests the possibility of developing drugs with specificity for parasite homologues. Experimental observations also suggest the possibility for drug selectivity. For example, *S. mansoni* muscle fibres show RyR-like Ca^2+^ responses that are activated by caffeine but insensitive to the classical RyR blockers, ruthenium red and neomycin [118]. Drugs with selectivity for insect RyRs have been described and these have been utilized to develop novel insecticides, which do not affect mammalian RyRs [119]. A variety of 1,4-dihydropyridines have also been shown to exhibit some selectivity for parasite Ca^2+^ influx channels over mammalian counterparts, and molecular determinants for their specificity have been defined [95]. These results suggest that rational design of therapeutic strategies targeted against parasite Ca^2+^ channels may be an attractive prospect and that newly discovered insecticidal RyR modulators, 1,4-dihydropyridines, or other Ca^2+^-permeable channel agonists and antagonists may represent attractive lead compounds.

This study presents the opportunity for cloning and functional characterization of putative intracellular Ca^2+^ channels and Ca^2+^ influx channels in pathogenic parasites, as well as the development of novel therapeutics. Future studies of parasite signalling pathways and completion of further parasite genome sequencing projects will lead to a deeper understanding of the presence and function of these channels in pathogenic parasites.

## Materials and Methods

### Genomes analyzed

The genomes of the following pathogenic parasites were examined (annotation release date in parentheses): *Plasmodium falciparum 3D7* (May 2007), *Plasmodium vivax SaI-1* (Jan 2008), *Plasmodium knowlesi strain H* (Apr 2008), *Toxoplasma gondii ME49* (May 2008), *Babesia bovis T2Bo* (Mar 2008), *Cryptosporidium hominis TU502* (Mar 2008), *Cryptosporidium muris RN66* (Oct 2008), *Cryptosporidium parvum Iowa II* (Nov 2006), *Leishmania braziliensis MHOM/BR/75/M2904* (Oct 2007), *Leishmania infantum JPCM5* (May 2007), *Leishmania major Friedlin* (Mar 2008), *Trypanosoma brucei TREU927* (Dec 2006), *Trypanosoma cruzi CL Brener* (Dec 2007), *Entamoeba histolytica HM-1:IMSS (Jun 2010), Giardia intestinalis* (Mar 2008), *Trichomonas vaginalis G3* (Mar 2008), *and Schistosoma mansoni* (Jul 2011). No homologues of any Ca^2+^ channels examined were identified in the genomes of *E. histolytica or G. intestinalis*. Other genomes analyzed include those of: *Homo sapiens* (Dec 2010), *Caenorhabditis elegans* (Apr 2011), *Drosophila melanogaster* (May 2011), *Paramecium tetraurelia* (Mar 2008) and *Ciona intestinalis* (Sept 2008).

### BLAST searches, alignments and topology analysis

BLASTP analysis was carried out in all cases, using the following human sequences (GenBank accession number in parentheses): full-length or pore sequences of IP_3_R1 (Q14643.2; pore region amino acids 2536-2608) or RyR1 (P21817.3; pore region residues 4877-4948), and full-length human sequences of TrpA1 (NP_015628; N-truncated sequence residues 765-end), TrpV1 (NP_061197; N-truncated sequence residues 430-end), TrpC1 (P48995; N-truncated sequence residues 350-end), CNGA1 (EAW93049; transmembrane sequence residues 200-420), CNGB1 (NP_001288), NMDA receptor NR1 (Q05586), NMDA receptor N2 (Q12879), AMPA receptor GRIA1 (P42261.2), kainate receptor GRIK1 (P39086), nAChR-alpha1 (ABR09427), purinergic receptor P2X4 (NP_002551.2), pannexin-1 (AAH16931), Orai1 (NP_116179.2), STIM1 (AAH21300), TPC1 (NP_001137291.1), TPC2 (NP_620714.2), TrpP1 (NP_001009944), TrpP2 (NP_000288), TrpM1 (NP_002411), TrpML1 (NP_065394), CatSper1 (Q8NEC5.3), mitochondrial uniporter (NP_612366.1) and Ca_v_1.2 (NP_955630.2). Sequences of the *S. cerevisiae* Ca^2+^ channel subunit Cch1 (CAA97244) and *Arabidopsis thaliana* TPC1 (AAK39554) were also used to search for parasite homologues.

BLASTP searches of the genomes of protozoan parasites, *H. sapiens*, *C. elegans*, *D. melanogaster* and *C. intestinalis* were carried out against the National Center for Biotechnology (NCBI) genomic protein databases. Searches of the *P. tetraurelia* genome were carried out using the BLASTP facility of ParameciumDB (v.1.59) (http://paramecium.cgm.cnrs-gif.fr) [120] to search the protein database (version 1, published 2006). The *S. mansoni* genome was searched using the BLASTP search facility of GeneDB (http://www.genedb.org/blast/submitblast/GeneDB_Smansoni) and *S. mansoni* BLASTP server twinscan2 peptide prediction facility (http://www.sanger.ac.uk/cgi-bin/blast/submitblast/s_mansoni) hosted by the Wellcome Trust Sanger Institute, UK [121]. Hits identified were then used as bait in further BLASTP searches (non-redundant protein database at NCBI) to identify corresponding annotated proteins. Several procedures ensured that hits were likely channel homologues. Firstly, the presence of multiple transmembrane domains was confirmed using TOPCONS [122]. Secondly, reciprocal BLASTP searches (non-redundant protein database at NCBI) were made, using the identified parasite hits as bait, and only proteins that gave the original mammalian protein family as hits were analyzed further. Thirdly, the presence of conserved domains was confirmed using the Conserved Domains Database (NCBI). Lastly, where possible pore homology was confirmed by sequence alignment using ClustalW2 (European Bioinformatics Institute). Where hits showed homology to more than one mammalian channel, BLASTP analysis against human sequences was used to identify the channel with greatest sequence similarity. Multiple sequence alignments were made using ClustalW2.1 and physiochemical residue colours are shown. For phylogenetic analysis, multiple sequence alignments were made with MUSCLE v3.7 (or ClustalW2.1 in the case of the lengthy IP_3_R/RyR homologues) using default parameters. After using GBLOCKS at low stringency to remove regions of low confidence, and removing gaps, Maximum Likelihood analysis was carried out using PhyML v3.0 (WAG substitution model; 4 substitution rate categories; default estimated gamma distribution parameters; default estimated proportions of invariable sites; 100 bootstrapped data sets). Phylogenetic trees were depicted using TreeDyn (v198.3). MUSCLE, GBLOCKS, PhyML and TreeDyn were all functions of Phylogeny.fr (http://www.phylogeny.fr/) [123].
